# Inhibitory Monoclonal Antibodies and Their Recombinant Derivatives Targeting Surface-Exposed Carbonic Anhydrase XII on Cancer Cells

**DOI:** 10.3390/ijms21249411

**Published:** 2020-12-10

**Authors:** Dovile Stravinskiene, Aiste Sliziene, Lina Baranauskiene, Vilma Petrikaite, Aurelija Zvirbliene

**Affiliations:** 1 Department of Immunology and Cell Biology, Institute of Biotechnology, Life Sciences Center, Vilnius University, Sauletekio al. 7, LT-10257 Vilnius, Lithuania; aiste.imbrasaite@gmc.vu.lt (A.S.); aurelija.zvirbliene@bti.vu.lt (A.Z.); 2 Department of Biothermodynamics and Drug Design, Institute of Biotechnology, Life Sciences Center, Vilnius University, Sauletekio al. 7, LT-10257 Vilnius, Lithuania; lina.baranauskiene@bti.vu.lt (L.B.); vilma.petrikaite@bti.vu.lt (V.P.); 3 Laboratory of Drug Targets Histopathology, Institute of Cardiology, Lithuanian University of Health Sciences, Sukileliu pr. 13, LT-50162 Kaunas, Lithuania

**Keywords:** carbonic anhydrase XII, cancer immunotherapy, monoclonal antibody, recombinant antibody, single-chain fragment variable, hybridoma

## Abstract

Monoclonal and recombinant antibodies are widely used for the diagnostics and therapy of cancer. They are generated to interact with cell surface proteins which are usually involved in the development and progression of cancer. Carbonic anhydrase XII (CA XII) contributes to the survival of tumors under hypoxic conditions thus is considered a candidate target for antibody-based therapy. In this study, we have generated a novel collection of monoclonal antibodies (MAbs) against the recombinant extracellular domain of CA XII produced in HEK-293 cells. Eighteen out of 24 MAbs were reactive with cellular CA XII on the surface of live kidney and lung cancer cells as determined by flow cytometry. One MAb 14D6 also inhibited the enzymatic activity of recombinant CA XII as measured by the stopped-flow assay. MAb 14D6 showed the migrastatic effect on human lung carcinoma A549 and renal carcinoma A498 cell lines in a ‘wound healing’ assay. It did not reduce the growth of multicellular lung and renal cancer spheroids but reduced the cell viability by the ATP Bioluminescence assay. Epitope mapping revealed the surface-exposed amino acid sequence (35-FGPDGENS-42) close to the catalytic center of CA XII recognized by the MAb 14D6. The variable regions of the heavy and light chains of MAb 14D6 were sequenced and their complementarity-determining regions were defined. The obtained variable sequences were used to generate recombinant antibodies in two formats: single-chain fragment variable (scFv) expressed in *E. coli* and scFv fused to human IgG1 Fc fragment (scFv-Fc) expressed in Chinese Hamster Ovary (CHO) cells. Both recombinant antibodies maintained the same specificity for CA XII as the parental MAb 14D6. The novel antibodies may represent promising tools for CA XII-related cancer research and immunotherapy.

## 1. Introduction

Antibodies, or immunoglobulins (Ig), are components of the humoral adaptive immune response, produced by B lymphocytes [1,2]. Since the introduction of hybridoma technology, monoclonal antibodies (MAbs) can now be produced in laboratories practically against any desired antigen [3]. Technologies of chimerization and humanization of murine MAbs led to the emergence of the first therapeutic recombinant antibodies (RAbs). RAbs possess reduced immunogenicity in humans and can be used for the treatment of cancer [4]. Recombinant intact immunoglobulin G (IgG) molecules have long half-life; therefore, they can accumulate at high concentration in the site of the targeted tumor despite their weak penetration. Besides, they can also initiate antibody-dependent cellular cytotoxicity (ADCC) providing an additional mechanism for tumor eradication [4,5]. Smaller antibody formats, such as single-chain fragment variable (scFv), may also be used for diagnostic and cancer therapy because they not only maintain the same antigen-binding activities as the intact IgG but also have better tumor penetration [3].

The use of RAbs for cancer immunotherapy is based on selecting the appropriate tumor-associated antigens, namely, proteins that are necessary for tumor development and survival [6,7]. Cell surface located antigens are accessible for antibodies and represent promising targets for antibody-based immunotherapy. Another mandatory feature of a suitable target is its homogeneous and high expression on cancer cells as well as its absence on normal tissues [8,9]. Carbonic anhydrases IX (CA IX) and XII (CA XII) are considered new targets for antibody-based immunotherapy as they fulfill the most requirements of cancer-related cellular markers. They both are transmembrane isozymes of the carbonic anhydrase (CA) family with extracellular active sites that catalyze the reversible hydration of carbon dioxide to bicarbonate [10,11,12]. CA IX and CA XII contribute to the adaptation of malignant cells to hypoxia and acidosis through the regulation of intracellular and extracellular pH. High CA IX expression is found in the kidney, lung, colon, breast, cervix, ovary, brain, and few other tumors, while CA XII is overexpressed in the kidney, gastric, colorectal, breast, and brain tumors [13,14].

Many attempts are made to produce highly specific therapeutic agents against cancer cells, selectively targeting CA IX and CA XII. Different classes of CA IX and CA XII inhibitors have been developed and their pharmacological evaluation is a key priority at the moment [15,16]. Small–molecule compounds—chemical inhibitors derived from acetazolamide, ethoxzolamide, benzenesulfonamide, salen, tetrahydrosalen, coumarin, and others—are under investigation for a selective CA IX and CA XII inhibition [13,17,18,19,20]. Insufficient selectivity and specificity are the major problems of small–molecule inhibitors. In contrast, MAbs are highly specific and selective for target antigens, therefore, they have the vast biological potential for cancer therapy [15].

First MAb G250 targeting the CA IX was used for the imaging of renal cell carcinoma in vivo, however, its murine format led to the emergence of human anti-mouse antibody (HAMA) [21,22]. A chimeric variant of G250 was used in numerous clinical trials, for both cancer imaging and therapeutic application; however, more studies are needed to confirm the efficacy of this antibody. As G250 does not inhibit the enzymatic activity of CA IX and initiates the lysis of CA IX-positive cells only through ADCC, new CA IX-specific antibodies were produced using phage-display technology [23]. Recombinant human scFvs can induce the internalization of extracellular membrane-bound CA IX into endosomes or inhibit its enzymatic activity [24]. Previous experiments on CA IX-positive cell membrane fragments, intact cells, and multicellular tissue-grows (spheroids) demonstrated the inhibitory potential of CA IX-specific Fab fragments [25].

Targeting CA XII is also beneficial for cancer therapy. It was shown that *CA9* knockdown inhibits tumor growth and decreases the number of cancer cells; however, the level of *CA12* mRNA is up-regulated. Combined silencing of both CA isoforms results in a lower xenograft tumor volume and higher cell death rate [11,26]. Recently, rat and murine MAbs inhibiting CA XII were developed and they are extensively investigated for antitumor activity [27,28,29]. Rat MAb 6A10 interferes with tumor cell growth in vitro and in vivo animal studies. The Fab fragment of this antibody was labeled with ^177^Lu or ^64^Cu to be used as an agent for radioimmunotherapy (RIT) or positron emission tomography (PET). It showed specific binding to CA XII on tumor cells in mice xenografts [30,31].

In the current study, we describe a novel collection of murine MAbs raised against the recombinant extracellular CA XII produced in a human cell line to maintain a glycosylation pattern similar to the native protein. One MAb inhibited the enzymatic activity of recombinant CA XII and recognized native CA XII on the surface of cancer cells. It was selected as a parental antibody to generate recombinant antibodies in two formats—scFv and scFv fused to human IgG1 Fc fragment (scFv-Fc), and their functional activity was investigated.

## 2. Results

### 2.1. Generation and Characterization of MAbs Specific to Human CA XII

To generate MAbs against human CA XII, mice were immunized with the recombinant extracellular domain of human CA XII expressed in human cell line HEK-293 (short antigen name—CA XII^HEK^). After fusion, more than 2500 viable hybrid clones were tested for the secretion of CA XII-specific antibodies and 40 of them were selected and further cloned by serial dilution to isolate a monoclonal cell population. After a few rounds of testing by an indirect ELISA and additional recloning steps, 24 stable hybridoma cell lines producing IgG antibodies against CA XII were obtained. The MAbs were characterized using ELISA, Western blot, and flow cytometry assays. Testing results are summarized in Table 1.

**Figure 1:**
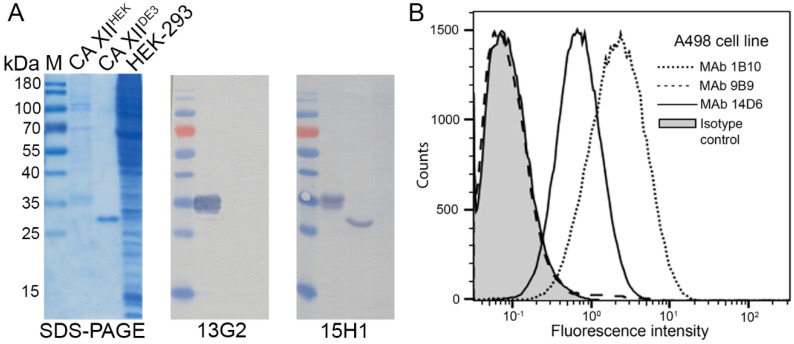
(**A**) Reactivity of the MAbs with SDS-denatured recombinant CA XII^DE3^ and CA XII^HEK^. Left panel: SDS-PAGE; right panels: immunoblot with MAbs 13G2 and 15H1. M—PageRuler Prestained Protein Ladder, 10 to 180 kDa (Thermo Fisher Scientific, Vilnius, Lithuania, 26616). The lysate of HEK-293 cells was used as a negative control. (**B**) Binding of the MAbs with native CA XII localized on the surface of live A498 cells was investigated by flow cytometry. The MAbs 1B10 and 14D6 reactive with cellular CA XII and the non-reactive MAb 9B9 are shown. Isotype control—the in house produced MAb of the relevant isotype raised against Porcine Parvovirus [32]. Secondary antibody: Alexa Fluor 488-conjugated Goat anti-Mouse IgG (H + L) (Thermo Fisher Scientific, A–11029).

The majority of MAbs (18 of 24) were of IgG1 subtype, two antibodies were IgG2a, and 4 of IgG2b subtype. All MAbs recognized linear CA XII epitopes as they were reactive with the antigen CA XII^HEK^ by Western blot after SDS-gel electrophoresis under denaturing conditions (SDS-PAGE) (Figure 1A). However, three of the MAbs (clones 9B9, 13G2, 20C4) were not reactive with recombinant non-glycosylated CA XII produced in *E. coli* (short name—CA XII^DE3^). Potentially, this indicates their specificity to the carbohydrate moiety of CA XII^HEK^, as demonstrated by ELISA and Western blot (Table 1, Figure 1A). In the next step, the apparent dissociation constants (K_d_) of the MAbs were determined by an indirect ELISA using plate-immobilized CA XII^HEK^. The K_d_ values calculated from three experiments ranged from 1.4 × 10^−8^ M to 7.7 × 10^−10^ M (Table 1), indicating high-affinity binding of the MAbs with CA XII^HEK^.

Recombinant CA I, CA II, CA III, CA IV, CA VA, CA VB, CA VI, CA VII, CA IX, CA XIII, and CA XIV [33] proteins were used to investigate the cross-reactivity of the MAbs by ELISA and Western blot (Table 1). Several MAbs were cross-reactive with recombinant CA I, CA II, CA VI, and CA XIII; however, the observed reactivity was near the limit of detection. Three MAbs of clones 9B9, 13G2, and 20C4 were strongly reactive with CA IV and CA IX produced in HEK-293 cells, which confirms MAb specificity for carbohydrate epitopes that were recognized irrespective of the protein carrier.

Flow cytometry assay was used to test MAb reactivity with CA XII localized on the cell surface. The majority of MAbs were able to bind the native CA XII on the surface of A498 cells (Figure 1B) and A549 cells (Table 1). Jurkat cells were used as a negative control as they do not express CA XII. The MAbs against the glycosylated part of CA XII^HEK^ (clones 9B9, 13G2, and 20C4) did not recognize cellular CA XII, suggesting different glycosylation patterns of the native cell-located and the recombinant CA XII^HEK^ proteins.

### 2.2. Inhibition of Recombinant CA XII by the MAbs

**Figure 2:**
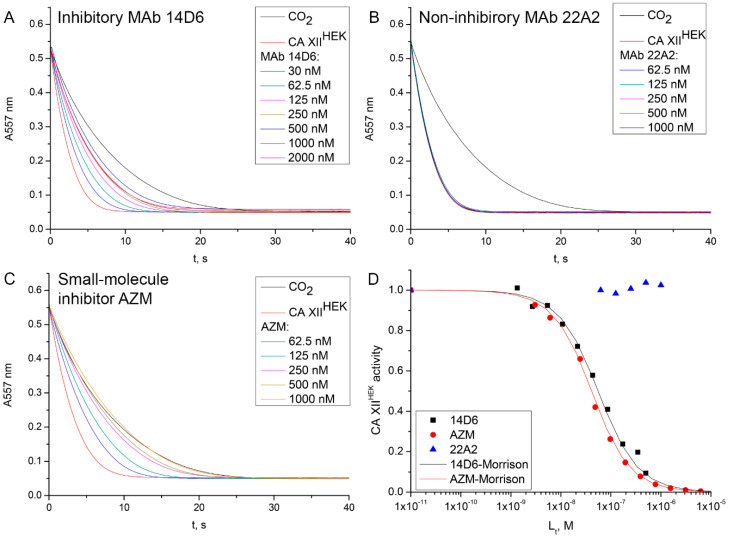
Inhibition of CO_2_ hydratase activity of CA XII^HEK^ by the MAbs. Raw data of dosing MAb 14D6 (**A**) and MAb 22A2 (**B**) are compared to the dosing of chemical inhibitor acetazolamide (AZM) (**C**). Black lines in panels A–C represent spontaneous CO_2_ hydration, while red ones—fully active enzyme. Panel (**D)** shows dosing curves, where symbols indicate the dependence of the observed enzyme activity on the concentration of the added ligand (MAb or small molecule compound, respectively) and lines represent the Morrison model used to determine inhibition constant. No inhibition of CA XII^HEK^ enzymatic activity is observed by adding MAb 22A2, while MAb 14D6 effectively inhibits the enzyme.

The MAbs reactive with cell surface-exposed CA XII were tested for potential inhibitory properties toward recombinant CA XII^HEK^ by stopped-flow CO_2_ hydratase activity method (Figure 2). The MAb 14D6 was distinguished from the others since it was the only MAb in this collection exhibiting high inhibition of the enzymatic activity of CA XII^HEK^. The calculated inhibition constant (~42 nM) was close to the inhibition constant of the classic CA inhibitor acetazolamide (AZM) (~32 nM).

### 2.3. MAb 14D6 Effect on A549 and A498 Cell Migration

**Figure 3:**
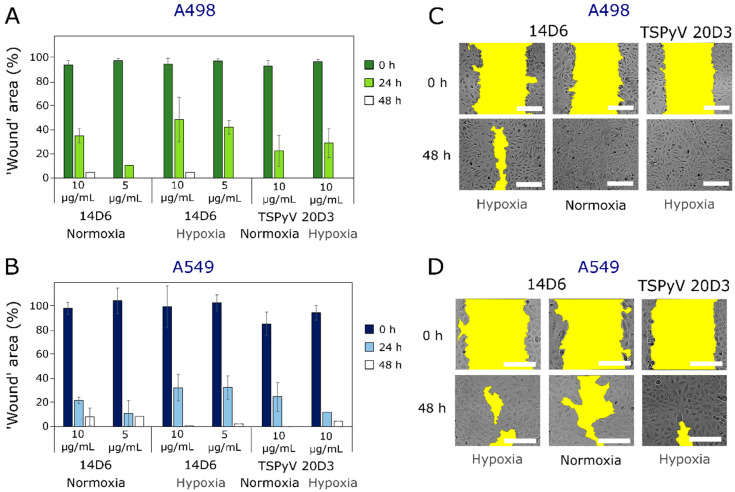
Effect of MAb 14D6 on human lung adenocarcinoma A498 (**A**) and A549 (**B**) cell migration under normoxia and hypoxia conditions. Photos of ‘wound’ area (marked in yellow) in A498 (**C**) and A549 (**D**) monolayer at 0 h and 48 h of treatment with antibody 14D6 and isotypic control TSPyV MAb 20D3. Scale bars indicates 100 µm.

Migrastatic effect of the MAbs on human lung cancer cell line A549 and human renal carcinoma cell line A498 was tested by ‘wound healing’ assay, also known as in vitro scratch assay [34]. The effect of MAb 14D6 was compared to the activity of the in-house isotypic control MAb 20D3 derived against an irrelevant antigen (trichodysplasia spinulosa-associated polyomavirus (TSPyV) capsid protein) [35]. MAb 14D6 showed greater inhibitory activity on A498 cell migration, especially in hypoxia conditions (Figure 3). Under hypoxia conditions, both concentrations of MAb 14D6 delayed the ‘healing of wound’ from 1.4 to 1.7 times compared to the isotypic control (Figure 3A). Moreover, MAb14D6 at a concentration of 10 µg/mL inhibited A498 cell migration to the ‘wound’ area also in normoxia after 48 h of incubation. Similarly, the migrastatic effect of MAb 14D6 on the A549 cell line was more expressed in hypoxia after 24 h of incubation at both tested concentrations (5 and 10 µg/mL) compared to the isotype control (Figure 3B). After 48 h, the cell migration inhibitory effect was more expressed in normoxia conditions, while this effect in hypoxia was practically negligible and comparable to the control.

### 2.4. Inhibitory Activity of MAb 14D6 in Three-Dimensional Cultures of A549 and A498 Cells

**Figure 4:**
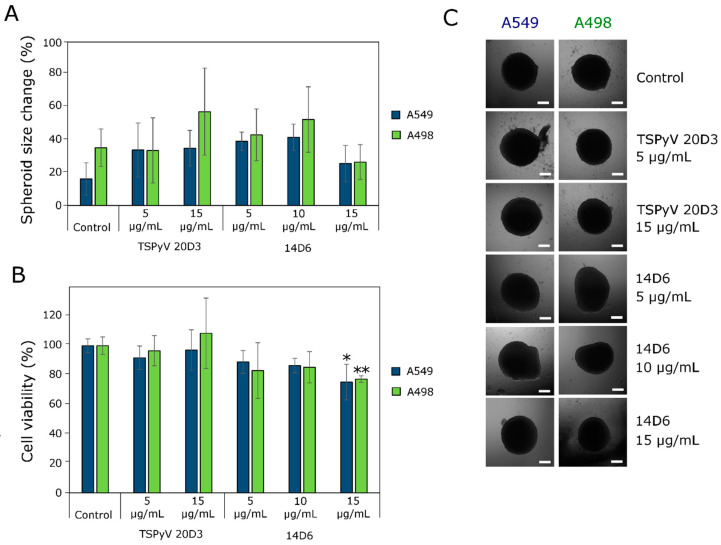
Effect of MAb 14D6 and isotype control (TSPyV MAb 20D3) on human lung adenocarcinoma A498 and A549 spheroids. (**A**) Spheroid size at the end of experiment. (**B**) Cell viability in A498 and A549 spheroids. (**C**) Photos of A549 and A498 tumor spheroids at the end of incubation with the MAbs. Scale bars indicated 100 µm. Asterisks (*) indicate *p* < 0.05 and (**) indicate *p* < 0.01 compared to the control (untreated spheroids).

The activity of MAbs in three-dimensional cultures (spheroids) was tested using a multicellular (cancer cells combined with fibroblasts) tumor spheroid model in vitro. MAb 14D6 did not have a significant impact on the size of spheroids (Figure 4A). The spheroids treated either with the MAb 14D6 or the irrelevant MAb (isotype control) seemed to be compact and tight at the end of the experiment (Figure 4C). However, the MAb 14D6 reduced the viability of spheroid cells as compared to the isotype control (Figure 4B). Spheroids treated with the isotype control possessed the same cell viability (ATP content) compared to the non-treated spheroids (control). In contrast, 14D6-treated spheroids were less viable: MAb 14D6 added at a concentration of 15 µg/mL reduced the ATP content by about 25% (A549 spheroid, *p* < 0.05) and 23% (A498 spheroids, *p* < 0.01), respectively.

### 2.5. Epitope Mapping and Competitive Properties of the MAbs

**Figure 5:**
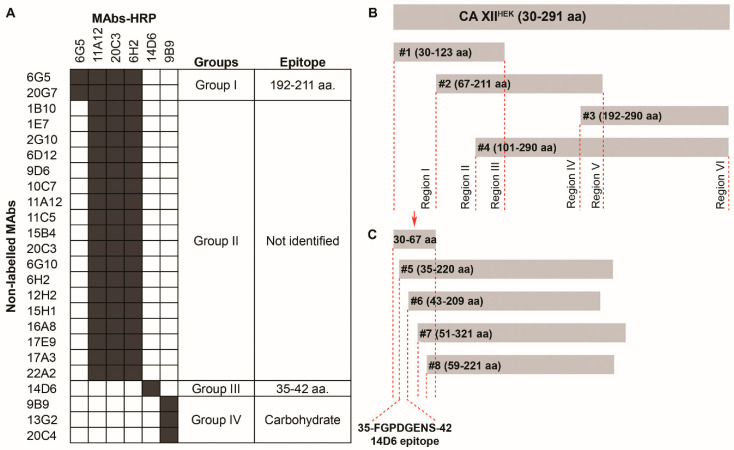
(**A**) Epitope localization and competitive binding properties of anti-CA XII MAbs. Black box—competitive MAbs; white box—non-competitive MAbs. (**B**,**C**) Schematic representation of overlapping His–fused CA XII fragments used for epitope mapping.

Six MAbs were selected based on their ability to recognize CA XII by different immunoassays (ELISA, Western blot, Flow cytometry) and were labeled with horseradish peroxidase (HRP) to be tested in a competitive ELISA. All conjugates were reactive with CA XII by direct ELISA and were used to determine the competing MAbs. In a competitive ELISA, undiluted hybridoma supernatants of 24 MAbs were incubated with plate immobilized CA XII^HEK^, allowing the formation of the antibody-antigen complex. Then MAb-HRP conjugates were added to the wells. All possible combinations of the non-labeled and HRP-conjugated MAbs were tested. The signal was observed if the non-labeled MAb and the conjugate were attached to different parts of the protein. Based on the competitive ELISA data, the MAbs were divided into four groups of non-competing MAbs (Figure 5A). It was assumed that the MAbs in the same group share the same or neighboring antigenic determinant, which is distinct from the epitopes recognized by the MAbs in other groups.

To localize the epitopes recognized by the MAbs, four overlapping His-fusion CA XII fragments (#1 V27–G123, #2 Y67–F211, #3 H192–S290, #4 P101–S290, Figure 5B) spanning the whole CA XII sequence were produced in *E. coli*. The expression of CA XII fragments in *E. coli* was confirmed by Western blot using the anti-His antibody (data not shown). From all tested MAbs, only 3 MAbs (clones 6G5, 14D6, 20G7) were reactive with CA XII fragments in Western blot (data not shown), thus allowing the localization of their epitopes (Figure 5A).

To summarize epitope mapping data, the MAbs of group I bind to the epitope localized in the CA XII region comprising of amino acids (aa) 192–211. These MAbs do not react with cellular CA XII. The MAbs of group II do not react with any of the overlapping fragments, suggesting that they recognize a conformation-dependent CA XII epitope that might require an intact molecule. These MAbs recognize CA XII on the cell surface. The MAbs of group IV presumably recognize the glycosylated part of recombinant CA XII. They do not react with CA XII fragments produced in *E. coli* bacteria, which do not proceed glycosylation. They also do not recognize CA XII on the cell surface by flow cytometry that indicates potentially different glycosylation of recombinant CA XII synthesized in HEK-293 and native CA XII expressed in other human cell lines. Only one MAb—clone 14D6—was assigned to group III as no other MAbs recognized the same epitope localized in the CA XII region comprising of aa 27–67.

**Figure 6:**
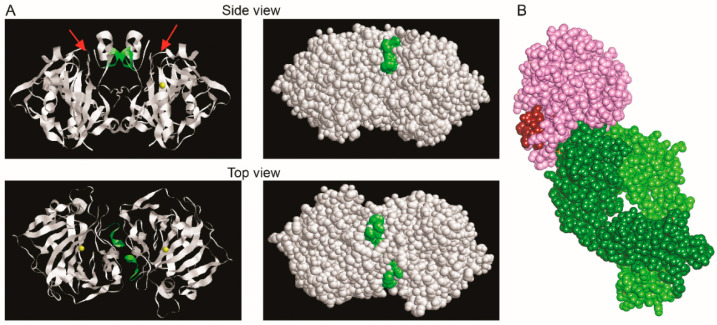
(**A**) Localization of the linear MAb 14D6 epitope within the CA XII dimer model (PDB ID: 1JD0, RasMol Version 2.7.5.2). Side and top view of molecule’s ribbon display (left panel) and molecular surface (right panel). Yellow represents zinc atoms, green—the sequence of the identified epitope, red arrows indicate the active site clefts, according to Whittington et al. 2001 [36]. (**B**) CA XII/Fab6A10 complex model (PDB accession code: 6RPS) with the position of epitope of MAb 14D6. CA XII is shown in pink, the epitope FGPDGENS of MAb 14D6—red, Fab6A10—light and dark green [37].

To specify the epitope of MAb 14D6, additional four N terminal truncated His-tagged overlapping CA XII fragments (#5 F35–R220, #6 W43–E209, #7 G51–N231, #8 D59–Y221, Figure 5C) were produced in *E. coli*. New fragments divided the identified CA XII region of aa 27–67 into five short sequences, each 5–8 aa in length. MAb 14D6 was reactive with fragment #5, thus indicating the CA XII sequence from aa 35 to 42 (FGPDGENS) as its epitope (Figure 5C). To identify its position within the CA XII molecule, a computer model of the CA XII catalytic domain based on Protein Data Bank (PDB) entry 1JD0 was developed. The MAb 14D6 epitope was localized on the surface of a dimeric CA XII molecule nearby its catalytic center (Figure 6A).

MAb 14D6 demonstrated properties necessary for the potential agent of therapeutic relevance: it was reactive with native cellular CA XII by flow cytometry and inhibited CA XII enzymatic activity. Consequently, we have selected MAb 14D6 for the generation of RAbs expecting to develop novel specific tools with potential antitumor activity.

### 2.6. Sequencing of the Variable Region of MAb 14D6

**Figure 7:**
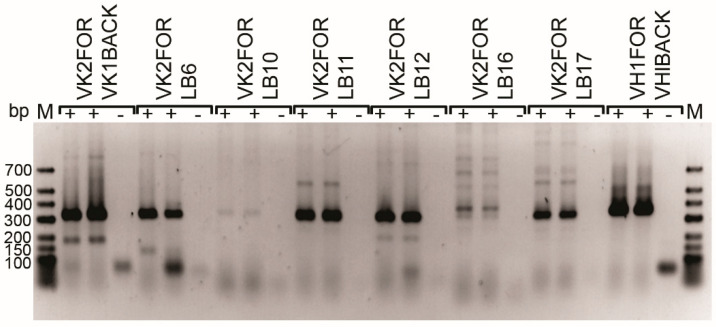
PCR amplification of MAb 14D6 H and L chain variable regions from the hybridoma cell line. PCR products were visualized by electrophoresis on 1% agarose gels. Line M—O’GeneRuler Low Range DNA Ladder 25–700 bp, ready-to-use (Thermo Fisher Scientific, SM1203); cDNA was used as a matrix (+) for PCR, and nuclease-free water was used as no template controls (–) for each set of primers.

As a first step for the generation of RAbs against CA XII, we have sequenced the variable (V) region of the inhibiting MAb 14D6. The variable Ig heavy (VH) chain and light (VL) chain regions of MAb 14D6 were cloned from the hybridoma cells. After total RNA isolation and single-strain cDNA synthesis, VH and VL regions were amplified by PCR using primers specific for mouse Ig heavy (H) and light (L) chains framework region (FR) 1 and isotype-specific region adapted from elsewhere [38,39,40]. PCR products were obtained using combinations of IgG1/MH1 and VH1FOR/VHlBACK primers pair for H chain and Kc/Mk, VK2FOR/VK1BACK, VK2FOR/LB6, VK2FOR/LB11, VK2FOR/LB12, and VK2FOR/LB17 (Figure 7) primers pairs for L chain. All amplified PCR products were cloned into a cloning vector and sequenced.

**Figure 8:**
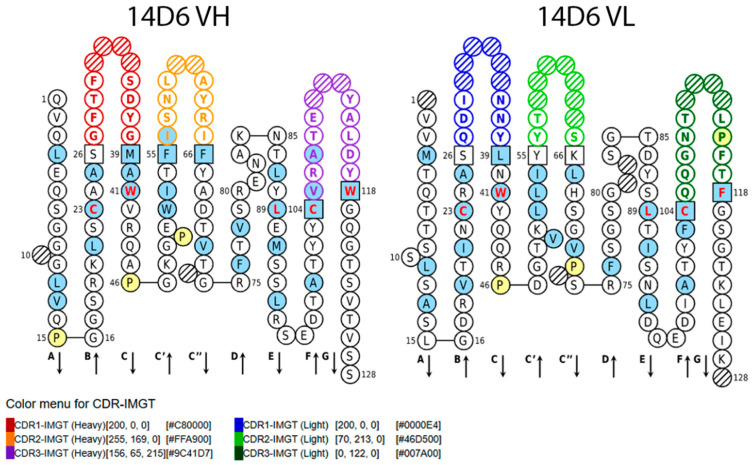
Standardized IMGT 2D graphical representations of potentially functional sequences of MAb 14D6 VH and VL chains. Positions in blue shows hydrophobic aa or tryptophan (W); Proline are shown in yellow; Red and bold letters indicate the five conserved aa positions of a V domain: 23 aa (1st cysteine), 41 aa (conserved tryptophan), 89 aa (hydrophobic aa), 104 (2nd cysteine), and 118 (hydrophobic aa or tryptophan). Squares indicate an anchor position. Hatched positions correspond to gaps according to the IMGT unique numbering for V domain. Arrows indicate the beta-strands and their direction. Sequences were analyzed with IMGT/DomainGapAlign tool [44,45], graphic was generated with IMGT/Collier-de-Perles [46,47].

Analysis with the IgBlast tool [41] and the analyzer IMGT/V–QUEST [42,43] revealed the cloned antibody sequences with occurred frameshifts, stop codons, deletions, or atypical aa residues, which were eliminated from further analysis. The quantity and position of the conserved cysteine residues were considered when selecting the appropriate sequence, thus obtaining properly rearranged and plausible productive sequences of the heavy chain (IgG1/MH1primers) and light chain (VK2FOR/LB12 primers). The positions and aa sequences of complementarity-determining regions (CDR) 1–3 and FR 1–4 of both chains were identified (Figure 8).

### 2.7. Production of 14D6-Derived scFv in E. coli

**Figure 9:**
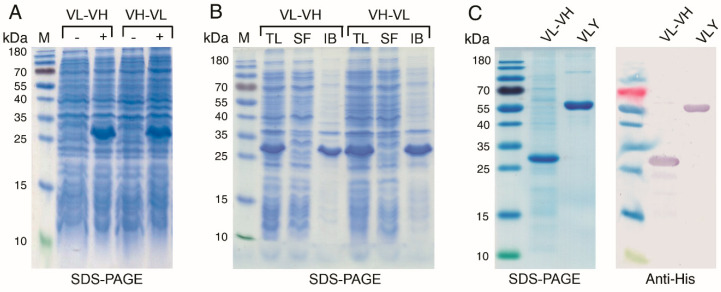
Production of 14D6-derived single-chain fragment variables (scFvs) in *E. coli*. (**A**) analysis of the lysates of *E. coli* expressing scFv variants VL–VH and VH–VL before (–) and after (+) induction; (**B**) scFv aggregated into insoluble inclusion bodies (IB), TL—total bacteria lysate, SF—soluble fraction; (**C**) Analysis of refolded and purified scFv VL–VH by SDS-PAGE and Western blot using anti-His antibody, recognizing His-tag of scFv and in-house produced recombinant vaginolysin (VLY), previously reported to contain His-tag as a positive control [48].

Two variants of anti-CA XII scFvs derived from hybridoma clone 14D6 and consisting of either VL–VH or VH–VL orientation linked with 20 aa long linker sequence (G_4_S)_4_ were constructed. Genes encoding both variants of scFvs were cloned into pET28a(+) vector for the expression of N-terminally 6xHis-tagged proteins in *E. coli* Tuner (DE3) strain. Proteins of approximately 30 kDa in size were highly expressed as insoluble inclusion bodies (Figure 9A,B). The inclusion bodies were solubilized in 7M GuHCl and refolded by adding CuSO_4_ followed by stepwise dialysis against dialysis buffer containing folding additive L-arginine and decreasing concentrations of GuHCl. The scFv variant VH–VL tended to aggregate during refolding, while the scFv VL–VH variant was successfully renatured. The refolded scFv VL–VH was purified by metal-chelate affinity chromatography and analyzed by SDS-PAGE under reducing conditions that revealed a homogeneous scFv of approximately 80% purity (calculated from WB figure with ImageJ software). This scFv was also recognized by anti-His secondary antibody in Western blot (Figure 9C).

### 2.8. Production of 14D6–Derived scFv-Fc in CHO Cell Line

**Figure 10:**
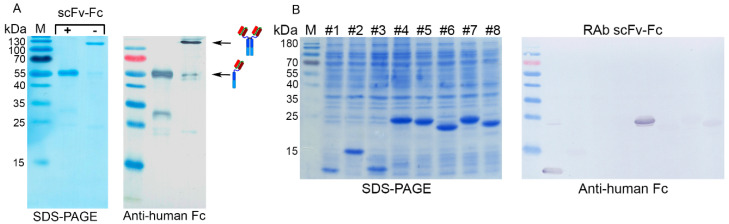
(**A**) Analysis of the Protein A–purified scFv-Fc by SDS–PAGE and Western Blot under reducing (+) and non–reducing (–) conditions. Anti-human Fc secondary antibody was used; (**B**) Analysis of the lysates of *E. coli* expressing overlapping fragments of CA XII with scFv-Fc and anti-human Fc secondary antibody by Western blot.

The scFv VL–VH derived from hybridoma clone 14D6 was used to develop the Fc-engineered scFv containing the Fc part of human IgG1. A stable CHO cell line secreting recombinant 14D6-derived scFv-Fc was established by transfecting adherent CHO cells with the newly constructed pFUSE-VL-(G_4_S)_4_-VH plasmid. The selection of RAb-expressing cells was conducted by adding 250 μg/mL zeocin into the growth medium. Antibody-producing single cell-derived subclones were isolated from the heterogeneous CHO cell pools using few rounds of limiting dilution. To evaluate the level of scFv-Fc production, CHO growth medium was analyzed by indirect ELISA using anti-human Fc polyclonal antibody. The scFv-Fc was purified using Protein A-Sepharose and further analyzed by Western blot under reducing and non–reducing conditions (Figure 10A). The reduced scFv-Fc migrated at ~56 kDa line, which is near the theoretically calculated protein size. The non-reduced scFv-Fc was ~112 kDa in size, indicating its homodimeric structure. The generated scFv-Fc was also analyzed on His-tagged overlapping CA XII fragments and showed the same binding profile with fragments #1 and #5 as the parental MAb 14D6 (Figure 10B).

### 2.9. Activity Testing of 14D6-Derived scFv and scFv-Fc

**Figure 11:**
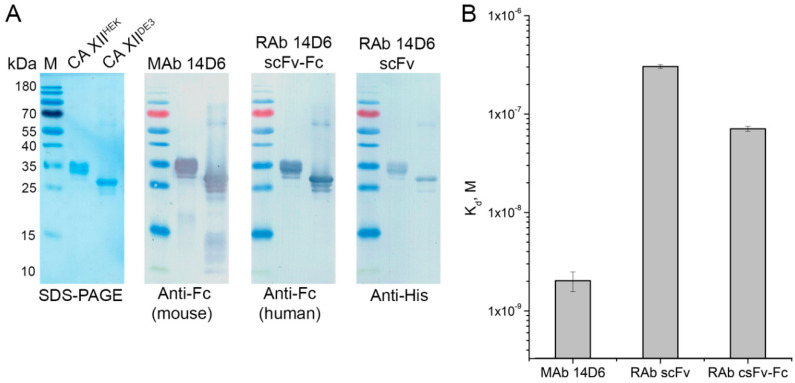
(**A**) Comparison of MAb 14D6, RAb scFv, and RAb scFv-Fc interaction with SDS-denatured recombinant proteins CA XII^HEK^ and CA XII^DE3^. (**B**) Apparent dissociation constants (K_d_, M) of MAb 14D6, RAb scFv, and RAb scFv-Fc determined by an indirect ELISA.

Both purified RAbs—scFv and scFv-Fc—were tested by Western Blot for the interaction with denatured recombinant CA XII^HEK^ and CA XII^DE3^ and were compared with the parental MAb 14D6. Both scFv and scFv-Fc were able to recognize the SDS-denatured CA XII^HEK^ and CA XII^DE3^, indicating the retained specificity of the parental MAb 14D6 (Figure 11A). Higher concentration of scFv (15 µg/mL) was required to develop the signal compared with scFv-Fc variant (10 µg/mL) or the parental MAb 14D6 (5 µg/mL). It is explained by different affinity (K_d_ values) of the RAbs and MAb 14D6. The determined K_d_ of the scFv-Fc (7.1 × 10^−8^ M) indicates four times higher affinity than scFv (K_d_ 3.0 × 10^−7^ M) and 35 lower than the parental MAb 14D6 (K_d_ 2.0 × 10^−9^ M) (Figure 11B).

## 3. Discussion

In our previous studies, we have applied different approaches and used different antigens to produce CA XII-specific MAbs. The collection of seven high-affinity MAbs against human CA XII was generated using an extracellular catalytic domain of human CA XII expressed in *E. coli* as an immunogen [49]. Two MAbs were raised against a synthetic peptide corresponding to the surface sequence of CA XII located near its catalytic center [29]. CA XII-specific MAbs from both collections were characterized as efficient tools for immunohistochemistry, Western blot, and ELISA. More importantly, peptide-derived antibodies had an inhibitory effect on recombinant CA XII. However, none of the MAbs recognized native CA XII on the surface of live cells [29,49].

In other studies, highly promising MAbs for cancer diagnostics and therapy were obtained by immunization of rats with CA XII-expressing A549 lung cancer cells [27]. MAb 6A10 was shown to be suitable for CA XII detection by flow cytometry and immunofluorescence. Moreover, it inhibited the catalytic activity of CA XII and the growth of tumor cells not only in vitro but also in vivo xenograft models [28]. Recently, a 6A10-derived Fab fragment developed in CHO cells was labeled with ^177^Lu or ^64^Cu and tested both in vitro and in vivo for PET or RIT applications [30,31]. Furthermore, a humanized antibody 4AG4 targeting CA XII was generated and showed blocking of CA XII enzymatic activity [50]. The results of these studies are promising and give expectations that MAbs or their derivates might be employed for cancer therapy shortly.

In the current study, the collection of MAbs against CA XII was derived using recombinant human CA XII expressed in human cell line HEK-293 (CA XII^HEK^). Recombinant proteins produced in HEK cells are likely to share features with natural human proteins in terms of post-translational modification and function [51]. HEK-293-derived antigen showed low immunogenicity in mice: the titer of CA XII-specific antibodies in the blood of immunized mice reached 1:1600. However, we succeeded to produce a collection of 24 stable hybridoma cell lines secreting IgG MAbs against CA XII. Novel MAbs were tested for various applications. They were reactive with the soluble CA XII by ELISA and the denatured CA XII by Western blot, showing their ability to recognize linear epitopes of CA XII. More importantly, 18 of 24 MAbs were reactive with native CA XII on human cancer cell lines of kidney (A498) and lung (A549) carcinoma, which is essential for the antibodies of potential therapeutic relevance. Subsequently, MAbs were examined for the ability to inhibit the enzymatic CO_2_ hydration reaction by the stopped-flow assay. MAb 14D6 was the only one MAb from the whole collection capable to inhibit CA XII hydration activity at the same scale as the classic small-molecule CA inhibitor acetazolamide.

In a ‘wound healing’ assay, MAb 14D6 was shown to possess migrastatic properties on lung adenocarcinoma cell line A549 and renal carcinoma cell line A498. This effect was more strongly expressed on A498 cell migration in hypoxia than normoxia conditions. This could be explained by the elevated expression of CA XII in hypoxia [52] and the inhibitory effect of MAb 14D6. The effect of MAb 14D6 on A549 cells was less expressed, especially after a longer incubation period (48 h). It could be explained by the fact that *CA9* expression in this cell line is much higher in hypoxia than in normoxia, and the amount of *CA12* is comparatively lower [53]. However, *CA9* is practically not expressed in the A498 cell line [52]. The revealed effect of MAb 14D6 on lung and renal cancer cell migration is consistent with the previously shown CA XII involvement in the cell migration process [54] and contributes to the stronger evidence to prove the importance of CA XII inhibition to avoid the formation of tumor metastasis [55].

Moreover, we showed that CA XII inhibition could contribute to the changes in the tumor spheroid microenvironment. Despite the MAb 14D6 did not significantly affect the spheroid growth, the viability of cells was reduced in MAb-treated spheroids of both A549 and A498 cultures. Battke et al. [27] also established the inhibitory effect of CA XII antibody 6A10 on A549 cell 3D cultures, grown on agarose. Cell viability in MAb 14D6-treated spheroids was lower compared to the isotype control. Interestingly, CA XII was also proposed as the target for multi-drug resistance by other research groups [56] due to its co-expression along with the P-glycoprotein on many tumor cells. This suggests that CA XII-specific antibodies could find their own niche and be exploited as multi-functional therapeutic and diagnostic agents in cancer therapy.

The analysis of epitopes revealed unique binding features of the MAb 14D6. It was non-competing with other MAbs as determined by a competitive ELISA, and no other MAbs were reactive with the same CA XII fragment produced in *E. coli*. We managed to identify the eight aa-long sequence (35–FGPDGENS–42) of CA XII required for the binding of MAb 14D6. According to Whittington et al., this surface-exposed sequence is near the active site cleft [36], thus, we assume that MAb binding can block the entry to the active site for substrate or alter the structure of CA XII, making it inactive. The epitope sequence of MAb 14D6 does not match the epitopes of previously described antibodies targeting CA XII. The MAbs 1D6 and 3C8 were deliberately developed against the CA XII surface sequence localized between aa 167–180 [29], another MAb 15A4 recognized linear sequence localized between aa 184–210 [49]. Immunization of rats with cancer cells resulted in the MAb 6A10 recognizing conformational epitope on the surface of CA XII. This was confirmed by resolving the crystallographic structure of CA XII/Fab6A10 complex [37]. It is important to highlight that the aa involved in CAXII/Fab6A10 interaction do not overlap with the epitope of MAb 14D6 (Figure 6B).

Based on these results, MAb 14D6 was considered an ideal candidate for the development of recombinant derivatives. Our strategy for the generation of RAbs targeting human CA XII was based on cloning and sequencing variable regions of immunoglobulin light and heavy chains (VL and VH, respectively) from hybridoma 14D6. Although other technologies such as antibody phage display may provide clinically relevant human recombinant antibodies, hybridoma-derived humanized antibodies still dominate in the market of biopharmaceuticals for cancer therapy [57]. Small-molecule inhibitors are also extensively tested, but the high specificity and affinity of MAbs give an advantage and are key features to evade off-target toxicity [58].

We generated two recombinant derivatives of MAb 14D6—scFv and scFv-Fc—containing human IgG Fc fragment. Tumor uptake of scFv is thought to be higher due to their small size. They are also cleared from the blood more rapidly as compared to full-length IgG. Although scFvs are lacking antibody effector function, they can be applied as immunotoxins when conjugated with drugs or radionuclides [3]. Moreover, scFv are components of chimeric antigen receptors for the recently approved T cell therapy [59,60]. The Fc-engineered scFv may provide a longer half-life, as well as IgG effector functions through the interaction of Fc and Fc-gamma receptors [61]. This shows a wide range of potential applications of murine-origin engineered antibody derivatives.

The specificity of the antibody is determined by 6 antigen-binding loops or CDRs on the light and heavy chains of immunoglobulin molecule that are hyper-variable in different antibodies [62]. In our study, the variable regions of light and heavy chains of 14D6 antibody were amplified using several primer pairs, including the degenerative primers mostly complementary for 5′ leader sequences. It is a tricky but crucial step, considering the diversity of antibody variable regions [63]. After the sequencing of different PCR products, only one light chain variable fragment obtained with VK2FOR/LB12 primers was found to be properly rearranged and productive. The heavy chain sequence was obtained with IgG1/MH1primers. These results suggest the importance of many diverse primers to increase the success rate of potentially correct variable sequences. Moreover, the CDR sequences of the MAb 14D6, which were determined in this study, are different from the CDRs of the previously described MAb 6A10 [37]. This explains the diverse properties of these MAbs.

The 14D6-derived scFv and scFv-Fc were expressed in *E. coli* and CHO cells, respectively. The scFv was less stable and tended to aggregate, therefore, its purification and refolding process was more complicated compared to scFv-Fc. The Fc-fusion proteins are straightforward to purify; however, more efforts are needed to obtain highly-secreting CHO clones and select a stable cell line. We generated one stable CHO clone secreting scFv-Fc of an appropriate dimeric structure. The activity analysis of both scFv and Fc-scFv by ELISA and Western blot confirmed their specificity for recombinant CA XII. However, their affinity was lower as compared to the parental MAb 14D6. The scFv-Fc was shown to bind the same CA XII epitope as the parental MAb 14D6.

In conclusion, we describe a novel collection of MAbs raised against cancer-associated enzyme CA XII. These well-characterized MAbs can be used for CA XII research and potentially for diagnostics. As CA XII might contribute to drug-resistant cancer phenotype [64], there is a growing demand for new reliable tools for CA XII detection. Cancer therapy based on CA XII targeting is also a promising trend, thus new formats of molecules interacting with CA XII contribute to the progress in this field [30,31,50]. From the MAb collection, we have selected the MAb 14D6 with desired features and generated its recombinant derivatives scFv and scFv-Fc that maintained the specificity of the parental MAb. These novel molecules can be further applied for a more comprehensive analysis and evaluation of their potential for the treatment of CA XII-related cancers.

## 4. Materials and Methods

### 4.1. Recombinant CA Antigens for Immunization, ELISA, and Western Blot

All recombinant CA proteins were kindly provided by Prof. D. Matulis and his colleagues (the Department of Biothermodynamics and Drug Design, Institute of Biotechnology, Life Sciences Center, Vilnius University, Lithuania).

The recombinant catalytic extracellular domain of human CA XII for immunization was produced in human cell line HEK-293 (CA XII^HEK^) and purified as described previously [49]. In brief, the pCEP4 vector (Thermo Fisher Scientific, V04450) was modified for the insertion of the *CA12* gene and secretion of recombinant CA XII^HEK^ protein into the cell growth medium. The expression of CA XII^HEK^ was performed using the FreeStyle Max 293 expression system (Thermo Fisher Scientific, K900010) according to the manufacturer’s recommendations. FreeStyle 293-F suspension cell culture was maintained in a 37 °C incubator with a humidified atmosphere of 8% CO_2_, on an orbital shaker platform rotating at 135 rpm. Recombinant CA XII^HEK^ was purified from the cell supernatant using a CA-affinity column containing p-(aminomethyl)benzenesulfonamide agarose (Merck KGaA, A0796, Darmstadt, Germany). The eluted CA XII^HEK^ was dialyzed against a storage buffer containing 10 mM Hepes (pH 7.5) and 50 mM NaCl and stored at −80 °C.

Expression of recombinant CA I and CA VI [65], CA II [66], CA III, CA IV, CA VA, CA VB, CA IX and CA XIV [67], CA VII and CA XIII [68], and CA XII (in *E. coli*, CA XII^DE3^) [69] was described previously. These CA isoforms were used as antigens for ELISA and Western blot.

### 4.2. Generation of Hybridomas

Fifty micrograms of recombinant CA XII^HEK^ were mixed with a complete Freund adjuvant (Merck KGaA, F5881) and injected subcutaneously into three female BALB/c mice (6–8 weeks old). The mice received subsequent immunizations of the same antigen dose without any adjuvant after 4 and 8 weeks from the first immunization. One month after the last immunization, blood serum samples were collected and tested for the highest CA XII^HEK^ specific antibody titer by an indirect ELISA. The selected mouse was boosted by subcutaneous injection with 50 μg of recombinant CA XII^HEK^ dissolved in PBS three days before the hybridization. Cell fusion was described in detail previously [70]. Spleen cells were fused with Sp2/0-Ag14 myeloma cells (American Type Culture Collection (ATCC), CRL-1581) using polyethylene glycol solution (Merck KGaA, P7181) and were grown in selective medium with HAT (hypoxanthine, aminopterin, thymidine) medium supplement (Merck KGaA, H0262). Hybrid cells producing antibodies against CA XII^HEK^ were recloned twice by limiting dilution. Hybridoma clones were cultivated in DMEM medium (Merck KGaA, F 0445) supplemented with 15% standardized fetal bovine serum (FBS superior) (Merck KGaA, S 0615), 2 mM L–glutamine (Merck KGaA, G7513), and 200 μg/mL gentamicin (Merck KGaA, A 2712) in a humidified atmosphere at 37 °C and 5% CO_2_.

Animal experiments were performed following the Lithuanian and European legislation (License No. LT-59-902, Permission No. 184 for the breeding of mice, and Permission No. 209 for the generation of polyclonal and monoclonal antibodies).

### 4.3. Enzyme-Linked ImmunoSorbent Assay (ELISA)

#### 4.3.1. Indirect ELISA

The antigen was immobilized in 96-well plates (Nerbe plus, 10–121–0000, Winsen, Germany) at 2–5 μg/mL in coating buffer (0.05 M sodium carbonate salt, pH 9.6) at 50 μL aliquot per well overnight at 4 °C. The plates were blocked with 200 μL/well of 2% Bovine Serum Albumin (BSA) solution in PBS for 1 h at room temperature (RT) and washed twice with PBS-T (0.1% Tween 20 in PBS) before incubation with antibody samples (blood samples of the immunized mice, hybridoma growth medium, CHO growth medium or purified MAb or RAb). Antibody samples were diluted in PBS-T according to experimental conditions and added to the wells (50–150 μL/well) for 1 h incubation at RT. The plates were washed 5 times with PBS-T and incubated with 50 μL per well of goat anti-mouse IgG (Bio-Rad, 1721011, Hercules, CA, USA) or Dako polyclonal rabbit anti-human IgG (Agilent, P0214, Santa Clara, CA, USA) conjugated to horseradish peroxidase (HRP) diluted 1:5000 or 1:2000, respectively, in PBS-T for 1 h at RT. The plates were rinsed 5 times with PBS-T and the enzymatic reaction was developed by adding 50 μL of NeA-Blue Tetramethylbenzidine (TMB) (Clinical Science Products, 01016–1, Mansfield, MA, USA) peroxidase substrate per well. After 5–10 min of incubation at RT, the reaction was stopped with 25 μL/well of 3.6% sulfuric acid and the optical density (OD) was measured at 450 nm (reference filter 620 nm) using Multiskan GO Microplate Spectrophotometer (Thermo Fisher Scientific, 51119200).

The isotypes of the MAbs were determined by ELISA using the Mouse Immunoglobulin Isotyping ELISA Kit (BD Biosciences, 550487, USA) according to the manufacturer’s protocol.

To determine the apparent dissociation constant (K_d_) of monoclonal and recombinant antibodies by indirect ELISA, antibodies were diluted to the concentration ranging from 1.9 × 10^−13^ M to 3.3 × 10^−8^ M to determine the half–titration point. The titration curve was used to calculate the apparent K_d_ using OriginPro 9.1 software’s (OriginLab, Northampton, MA, USA) Sigmoidal Fit gadget.

#### 4.3.2. Competitive ELISA

Antigen coating, blocking, and washing were performed as described above. After blocking and washing, plates were incubated with undiluted hybridoma growth medium at 50 μL aliquot per well for 1 h at RT. Fifty microliters of MAbs-HRP conjugates (1:100–1:1000 dilution) were added per well and incubation was continued for 1 h. The enzymatic reaction was developed and measured as described above.

#### 4.3.3. Direct ELISA

Antigen coating, plate blocking, washing, signal development, and measurement were the same as described in indirect ELISA. Samples of MAb-HRP conjugates were diluted from 1:100 to 1:12800 with PBS-T buffer and incubated for 1 h at RT.

### 4.4. Protein Electrophoresis and Western Blot

Proteins (1 μg in case of purified proteins, 20–30 μg in case of cell lysate) were mixed with Lane Marker Reducing Sample Buffer (5X) (Thermo Fisher Scientific, 39000) and heated at 100 °C for 5 min and loaded on 9–12.5% sodium dodecyl sulfate-polyacrylamide gel (SDS-PAG). After separation (Minigel-Twin 846–010–100 and Multigel 846-010-200, Biometra, Göttingen, Germany) under denaturing conditions, proteins were transferred (Fastblot B33 846-014-100 Biometra) to a polyvinylidene difluoride (PVDF) membrane (Carl Roth, T830.1, Karlsruhe, Germany). The gels after SDS-PAG electrophoresis (SDS-PAGE) were stained using PageBlue Protein Staining Solution (Thermo Fisher Scientific, 24620). The PVDF membranes were blocked at RT for 1 h or overnight at 4 °C in blocking buffer: PBS containing 0.05% Tween 20 and 2–3% powdered milk (Carl Roth, T145). The membranes were incubated in a blocking buffer containing primary antibodies (hybridoma supernatant, cell growth medium, purified monoclonal or recombinant antibodies) positive and negative controls for 1 h at RT. The membranes were rinsed 5 times with 0.05% Tween 20 in PBS and placed into blocking buffer containing secondary goat anti-mouse IgG (Bio-Rad, 1721011, USA) (1:4000 dilution) or Dako polyclonal rabbit anti-human IgG (Agilent, P0214, USA) (1:1000) antibodies conjugated to HRP for 1 h at RT. Membranes were then washed 5 times with 0.05% Tween 20 in PBS and the signal was developed using TMB Liquid Substrate System for Membranes (Merck KGaA, T0565) ready-to-use solution.

### 4.5. Flow Cytometry

The ability of MAbs to recognize native CA XII on the cell surface was analyzed by flow cytometry assay using CA XII expressing human cell lines A–498 (human kidney carcinoma, ATCC, HTB-44) and A-549 (human lung adenocarcinoma, ATCC, CCL-185). As a negative control, Jurkat cells (human acute T cell leukemia, ATCC, TIB-152) that do not express CA XII were used [49]. Cells were cultivated in RPMI-1640 liquid medium with stable glutamine (Merck KGaA, FG 1215) supplemented with 10% standardized fetal bovine serum (FBS superior) (Merck KGaA, S 0615), 2 mM L-glutamine (Merck KGaA, G7513), and 200 μg/mL gentamicin (Merck KGaA, A 2712) in a humidified atmosphere at 37 °C and 5% CO_2_ to approximately 90% confluence. Cells were harvested by centrifugation or detached with trypsin (Merck KGaA, L 2153), washed with rinse buffer (PBS containing 1% BSA and 0.1% sodium azide), and 10^6^ cells per test were incubated with undiluted hybridoma supernatant at 4 °C for 30 min. After incubation, cells were washed with rinse buffer and stained for 30 min in the dark with Goat anti-Mouse IgG (H+L) Highly Cross-Adsorbed Secondary Antibody, Alexa Fluor 488 (Thermo Fisher Scientific, A-11029) diluted 1:200 in rinse buffer. Before analysis, cells were washed and suspended in Stain Buffer (BD Bioscience, 554657, San Jose, CA, USA). Flow cytometry was performed using CyFlow Space flow cytometer (Sysmex Partec, CY-S-3001, Goerlitz, Germany). Flow cytometry data were analyzed using FlowJo (FlowJo, Version 10, LLC, Ashland, OR, USA) analysis platform.

### 4.6. Purification of MAbs and scFv-Fc

MAbs (different mouse IgG subtypes) were purified from hybridoma supernatants, and the scFv-Fc (human IgG1) fusion protein was purified from CHO supernatant using rProtein A Sepharose Fast Flow (GE Healthcare Life Sciences, 17127901, Chicago, IL, USA). The supernatants were diluted in binding buffer (1.5 M glycine, 3 M NaCl, pH 8.9) at 1:2 ratio. Before loading the samples, the column was equilibrated with 10 column volumes of binding buffer. The antibody solution was applied on the column and subsequently washed with 5 column volumes of binding buffer. Elution was performed with 100 mM glycine, pH 3. The eluates were collected and pH neutralized by adding 1/10 volume of 1M Tris-HCl, pH 8.2. Collected fractions were analyzed by SDS-PAGE and antibody-containing fractions were pooled and dialyzed against PBS. Antibody concentrations were determined with NanoDrop 2000 spectrophotometer (Thermo Fisher Scientific, ND-2000) by measuring the absorbance at OD_280_.

### 4.7. Conjugation of MAbs with Horseradish Peroxidase

HRP (Merck KGaA, P8375) was linked to MAbs by a periodate oxidation reaction as described previously [71]. In brief, 5 mg of HRP was dissolved in 1 mL distilled water and mixed with 0.25 mL freshly prepared 0.2 M sodium periodate (Merck KGaA, 71859) for 20 min in dark at RT. After incubation, HRP was loaded onto Sephadex G-25 in PD-10 Desalting Columns (GE Healthcare Life Sciences, 17085101) to exchange buffer to 1 mM sodium acetate (pH 4.5). MAbs were dialyzed against 0.01 M sodium carbonate buffer (pH 9.5) and mixed with HRP at ratio 1:1 (*w/w*). The final pH was approximately adjusted to 9.5 with 0.2 M sodium carbonate and the MAbs-HRP mixture was incubated for 2 h at RT in dark with occasional shaking. Freshly prepared 4 mg/mL sodium borohydride solution was added to the final concentration of 0.2–0.4 mg/mL to stabilize the conjugate and was incubated for 2 h at 4 °C. The MAb-HRP conjugate was then dialyzed against PBS and stabilized by adding 2% BSA Fraction V (Merck KGaA, 05482) and 50% glycerol and stored at –20 °C. The activity of conjugates was tested by a direct ELISA. The dilution of conjugates for other experiments was selected from the titration curve corresponding to the OD_450_ of approximately 1.0.

### 4.8. CA XII Inhibition Assay

Khalifah’s pH Indicator Stopped–Flow method [72] was used to determine the inhibitory activity of the MAbs. Recombinant CA XII and the purified MAbs were used for the measurements with SX.18MV-R spectrophotometer (Applied Photophysics, Leatherhead, UK) at 25 °C temperature. Samples contained 25 mM Hepes at pH 7.5, 50 mM NaCl, 20 nM CA XII, and different concentrations of MAbs (ranging from 0–2 μM) or CA inhibitor acetazolamide as a positive control. The substrate solution was prepared by bubbling Milli–Q water with CO_2_ at 25 °C for one hour to obtain ~17 mM CO_2_ final concentration during experiments. To detect the change of pH during reaction progression, 30 μM Phenol Red was used as an indicator (absorbance maximum at 557 nm). The CA-catalyzed CO_2_ hydration reaction was compared to the uncatalyzed (spontaneous) CO_2_ hydration. CA XII and MAb were incubated for 1 h at 25 °C, allowing the formation of the enzyme–antibody complex before the measurements. The inhibition constants were determined using the Morrison equation [73,74].

### 4.9. Sequencing of Antibody Variable Regions

To determine the aa sequence of MAb 14D6 heavy chain and light chain variable regions (VH and VL, respectively), total RNA from hybridoma cells (3 × 10^6^) was first isolated using GeneJET RNA Purification Kit (Thermo Fisher Scientific, K0731) according to manufacturer’s protocol. Single-strain cDNA was synthesized using random hexamers with RevertAid H Minus First Strand cDNA Synthesis Kit (Thermo Fisher Scientific, K1631). VH and VL were amplified by PCR using a set of primers (Table 2) specific for mouse Ig heavy and light chains as described previously [75]. PCR amplification was performed using DreamTaq PCR Master Mix (2X) (Thermo Fisher Scientific, K1071), containing 2 μL of cDNA reaction mix and 0.2 μM of primer (primers were paired in different combinations). The PCR was performed in Applied BiosystemsVeriti 96-Well Thermal Cycler (Thermo Fisher Scientific, 4375786) according to these conditions: an initial reaction for 3 min at 94 °C, 30 cycles for 1 min at 95 °C, 1 min at 55 °C, 1 min at 72 °C, and final extension for 10 min at 72 °C. PCR products were then cloned into cloning vector pJET1.2/blunt using CloneJET PCR Cloning Kit (Thermo Fisher Scientific, K1231). Five clones of each fragment were sequenced using BigDye Terminator v3.1 Cycle Sequencing Kit (Thermo Fisher Scientific, 4337455) and Applied Biosystems 3130xl genetic analyzer (Thermo Fisher Scientific, 4359571). The nucleotide sequences of the PCR products were determined and translated to a protein sequence using the Translate tool (ExPASy proteomics server: https://www.expasy.org/). VL and VH sequences were analyzed with the IgBlast tool of the National Center for Biotechnology Information (NCBI, https://www.ncbi.nlm.nih.gov/igblast/). The CDRs were determined using an integrated alignment tool for the immunoglobulin and T cell receptor nucleotide sequences IMGT/V-QUEST of the international ImMunoGeneTics information system IMGT/V-QUEST (IMGT: http://www.imgt.org/IMGT_vquest/vquest).

### 4.10. Construction of Expression Vectors for 14D6-Derived scFv and scFv-Fc

The cloned VL and VH sequences were used to construct expression plasmid for 14D6-derived scFv as described previously [76]. First, new primers were designed to insert restriction sites of NheI and SalI for ligation into expression vector pET28a(+) (Merck KGaA, 69864) and the linker between VL and VH, encoding (G_4_S)_4_ (Figure 10). For the ligation of the linker sequence, BamHI restriction sites were added to both fragments. After PCR (reaction components, concentrations, and conditions were the same as described above), DNA fragments were ligated into cloning vector pJET1.2/blunt using CloneJET PCR Cloning Kit. Sequencing and restriction analyses were performed to ensure the proper state of the sequences. A plasmid containing VL fragment was linearized and the VH insert was digested using BamHI and XbaI restriction endonucleases (Thermo Fisher Scientific). VH fragment was then introduced into the linearized pJET1.2-VL plasmid by ligation resulting in a construct VL-(G_4_S)_4_-VH, which was later recloned into the expression vector pET28a(+) throughout NheI and SalI (Thermo Fisher Scientific) restriction sites (Figure S1).

To construct expression plasmid for 14D6-derived scFv-Fc, VL-(G_4_S)_4_-VH encoding sequence was reamplified from pJET1.2-VL-(G_4_S)_4_-VH using new primers with restriction sites of restriction endonucleases EcoRV and NcoI (Thermo Fisher Scientific). After PCR (reaction components, concentrations, and conditions were the same as described above), the cloned DNA fragments were ligated into the pFUSE-hIgG1-Fc2 plasmid (InvivoGen, San Diego, CA, USA) designed for the expression of secreted Fc–fusion proteins.

### 4.11. Generation of Recombinant scFv and scFv-Fc

To induce recombinant scFv expression, the plasmid pET28a(+)-VL-(G_4_S)_4_-VH was transformed into *E. coli* Tuner (DE3) (Merck KGaA, 70623) competent cells grown in LB broth medium (Merck KGaA, L3022) supplemented with 0.1 mg/mL kanamycin (Merck KGaA, K0129) and 0.1 mM IPTG (isopropyl-b-D-thiogalactopyranoside) (Thermo Fisher Scientific, 15529019) for 3 h at 37 °C shaker. The transformed cells were harvested, centrifuged at 10,000 rpm for 15 min at 4 °C, and disrupted by sonication (SONOPULS Ultrasonic homogenizer, Bandelin) in lysis buffer (0.1 M Tris-HCl pH 7, 5 mM EDTA, 0.1% Triton X-100, 1 mM phenylmethylsulfonyl fluoride (PMSF), 50 mM DTT). The inclusion bodies were enriched by centrifugation, followed by washing with PBS supplemented with 2% SDS. The supernatant (soluble fraction) and the inclusion bodies (insoluble fraction) were then analyzed by 12.5% SDS-PAGE under reducing conditions.

The secreted recombinant 14D6-derived scFv-Fc was generated by establishing a stable Chinese Hamster Ovary (CHO) cell line. Adherent CHO (ATCC, CCL-61) cells were cultured in a medium mixture consisting of a 1:1 ratio of Gibco Ham F-12 (Thermo Fisher Scientific, 11765054) and HyClone DMEM (ThermoFisher Scientific, SH30243) supplemented with 10% standardized FBS (FBS superior) (Merck KGaA, S 0615), 2mM L-glutamine (Merck KGaA, G7513), and 200 μg/mL gentamicin (Merck KGaA, A 2712) in a humidified atmosphere at 37 °C and 5% CO_2_. CHO cells were transfected by seeding 6.5 × 10^4^ cells in 900 μL medium without serum in 24 well plates (TPP, 92024) one day before transfection. On the day of transfection, 2 μg pFUSE-VL-(G_4_S)_4_-VH plasmid was combined with 4 μL of transfection reagent TurboFect (Thermo Fisher Scientific, R0531) and incubated for 20 min at RT. After incubation, the transfectant–DNA mixture was added to pre-seeded CHO cells and incubated for 72 h in an incubator. Antibody expressing cells were selected by adding 250 μg/mL zeocin (Thermo Fisher Scientific, R25001). Single cell clones were isolated from the heterogeneous CHO-mAb cell pools by limiting dilution. The productivity of clones was analyzed by indirect ELISA. The recloning by limiting dilution was repeated twice to isolate high-producing single cell-derived subclones. The expression of recombinant 14D6-derived scFv-Fc was confirmed by indirect ELISA and Western blot.

### 4.12. Refolding and Purification of scFv

To produce a sufficient quantity of purified scFv for its activity testing, 33 mL of transformed overnight *E. coli* Tuner (DE3) culture was transferred to 1 L LB broth medium (0.1 mg/mL kanamycin) and shaken (200 rpm) at 37 °C until OD_600_ = 0.8. To induce scFv production, the IPTG (final concentration 0.1 mM) was added to the culture, and shaking was continued for 3 h. The cells were collected by centrifugation and disrupted by sonication as described above. The inclusion bodies were washed with washing buffer (1M NaCl, 0.1% Tween 80) and solubilized in 7 M GuHCl, 10 mM Tris-HCl (pH 7, 4 °C) buffer (1 g of biomass suspended in 10 mL buffer) overnight at 4 °C. Prior to refolding, protein concentration was measured by Bradford assay and the sample was diluted in 6 M GuHCl, 10 mM Tris-HCl (pH 7, 4 °C) until 1 mg/mL final protein concentration. To refold the scFv, CuSO_4_ solution at a final concentration of 20 μM was added and the mixture was incubated for 2 h at RT. The reaction was stopped by adding EDTA until 10 mM final concentration. The refolded proteins were dialyzed against the dialysis buffer (25 mM Tris–HCl, 0.25 M Na_2_SO_4_, pH 7 at 4 °C) containing stepwise decreasing concentrations of GuHCl: 3.5 M, 2 M, 1 M (adding 400 mM L-arginine), 0.5 M (adding 400 mM L-arginine). Each step of dialysis was performed overnight. The 14D6-derived scFv was then purified by affinity chromatography on Chelating Sepharose Fast Flow (GE Healthcare Life Sciences, 17057501) according to the manufacturer’s instructions. The recovery of proteins bound to the column was achieved by a competitive stepwise elution using an increasing concentration of imidazole (50 mM, 100 mM, 200 mM, and 500 mM). The eluted fractions were analyzed by SDS-PAGE and those containing the 30.1 kDa size protein were pooled and dialyzed against PBS. The concentration of the purified scFv was measured spectrophotometrically at 280 nm according to the calculated extinction coefficient (43.1 × 10^3^ M^–1^cm^–1^) and the molecular weight (30.1 kDa) of scFv. The purity and immunoreactivity of the scFv were analyzed by an indirect ELISA, SDS-PAGE, and Western blot using HRP-labeled anti-His antibody (HIS.H8) (Thermo Fisher Scientific, MA1-21315-HRP).

### 4.13. Cell Culturing for Functional Assays

Human lung adenocarcinoma cell line A549 and human renal carcinoma cell line A498 were obtained from the American Type Culture Collection (ATCC, Manassas, VA, USA). Cells were grown in DMEM Glutamax medium (Gibco, Carlsbad, CA, USA) containing 10% fetal bovine serum and 1% antibiotic mixture (10,000 U/mL penicillin and 10 mg/mL streptomycin; Gibco). Human foreskin fibroblasts CRL-4001 (ATCC) were kindly provided by Dr. Ramūnas Valiokas (Center for Physical Sciences and Technology, Department of Nanoengineering). Fibroblasts were grown in Medium 106 with Low Serum Growth Supplement (Gibco). All cells were incubated at 37 °C in a humidified atmosphere containing 5% CO_2_.

### 4.14. Testing the Inhibitory Activity of the MAb 14D6 on A549 and A498 Cell Migration by ‘Wound Healing’ Assay

A549 and A498 cells were seeded in 24-well plates at a density of 6 × 10^4^ cells per well and incubated for 48 h at 37 °C in an atmosphere containing 5% CO_2_. Then, a scratch was made in the cell monolayer using a sterile 100 µL pipette tip. The cells were washed gently with PBS and a fresh medium containing 5 and 10 μg/mL of antibodies was added. MAb 20D3 against TSPyV was used as isotypic control. Hypoxia conditions were induced by adding 240 µM of Cobalt chloride into the medium. The cells were incubated at 37 °C in a humidified atmosphere containing 5% CO_2_.

‘Wounds’ were captured at the intervals of 0 h, 24 h, and 48 h from scratch under phase-contrast microscopy at a ×4 magnification. The wound area was analyzed using ImageJ program (National Institutes of Health, Bethesda, MD, USA).

### 4.15. Testing the Inhibitory Activity of the MAb 14D6 in Cancer Spheroids

Spheroids were formed by the magnetic 3D Bioprinting method from A549 and A498 cells mixed with human fibroblasts (1:1) to better imitate the tumor microenvironment. The procedure was described elsewhere [77]. Briefly, cells were incubated with nanoparticles NanoShuttle (Nano3D Biosciences Inc., Houston, TX, USA) for 10 h at 37 °C in a humidified atmosphere containing 5% CO_2_. Then, cells were plated in ultra-low attachment 96-well plates (1000 cancer cells and 1000 fibroblasts/well), placed on a magnetic drive, and incubated for 72 h, until spheroids were formed. Then, the medium was replaced by the fresh one containing 5 μg/mL, 10 μg/mL, and 15 μg/mL of antibodies. The phase-contrast images of spheroids were taken using an inverted microscope Olympus IX73 (Olympus, Tokyo, Japan). The effect of antibodies on cancer spheroids was evaluated by their differences in size, measuring the change of diameter with ImageJ program (National Institutes of Health).

The effect on cell viability was evaluated by an ATP-based luminescent cell viability assay. After 6 days of incubation with antibodies, the number of living cells was determined by the ATP-luminescent-based cell viability kit (CellTiter-Glo^®^, Promega, Madison, WC, USA), according to the manufacture’s protocol. Each experiment was performed at least in triplicate. Spheroids cultured in growth medium without antibodies or only medium served as negative and positive controls, respectively. The luminescence was measured in a Tecan Infinite 200 microplate reader (Tecan Trading AG, Männedorf, Switzerland).

### 4.16. Production of His-Tagged CA XII Fragments for Epitope Mapping

To primarily locate the MAb epitopes, the extracellular domain (25–301 aa) of CA XII was divided into 4 overlapping fragments of different lengths (#1–#4, Table 3). DNA sequences encoding these fragments were amplified from mRNA isolated from A498 cells by PCR using primer pairs shown in Table 3. PCR products were cloned into bacterial expression vector pET-16b (Merck KGaA, 69662) carrying the N terminal His-Tag sequence. The plasmids were transformed into *E. coli* Tuner (DE3) competent cells and the expression of fragments was induced as described above. The lysates of bacterial samples collected before and after induction were analyzed by SDS-PAGE. The expression levels of His-tagged fragments were evaluated by Western blot using HRP-labeled 6x-His Tag Monoclonal Antibody (HIS.H8), (Thermo Fisher Scientific, MA1-21315-HRP). The lysates were then tested with MAbs and RAbs by SDS-PAGE and Western blot.

For fine epitope mapping of 14D6 MAb and RAb, a new set of overlapping fragments was produced. The CA XII region V27–Y67, which was recognized by 14D6 MAb, was divided into 4 overlapping fragments (#5–#8, Table 3). New primers were designed and His-tagged proteins were produced as described above.

## 5. Patents

The authors D.S. and A.Z. are inventors on a patent of New recombinant antibody against human carbonic anhydrase XII, which was issued by The State Patent Bureau of the Republic of Lithuania and published on 10 November 2016 (No. 6331 B).

## Figures and Tables

**Table 1:** Summarized data on the characterization of the monoclonal antibodies (MAbs) raised against carbonic anhydrase XII^HEK^ (CA XII^HEK^) using ELISA, Western blot (WB), and flow cytometry (FC) assays.

MAb Clone	Isotype	ELISA, WB		Cross-Reactivities (ELISA, Wb)	FC			**K_d_, M**
		CA XII^HEK^	CA XII^DE3^		A498	A549	Jurkat	
1B10	IgG1	+	+	–	+	+	–	2.4 × 10^−10^
1E7	IgG2b	+	+	CA XIII	+	+	–	1.4 × 10^−9^
2G10	IgG2b	+	+	CA XIII	+	+	–	5.3 × 10^−10^
6D12	IgG1	+	+	–	+	+	–	1.1 × 10^−9^
6G5	IgG2a	+	+	CA I, CA II	–	–	–	ND *
6G10	IgG1	+	+	–	+	+	–	1.2 × 10^−9^
6H2	IgG1	+	+	–	+	+	–	1.7 × 10^−8^
9B9	IgG1	+	–	CA IV, CA IX	–	–	–	ND *
9D6	IgG1	+	+	–	+	+	–	2.3 × 10^−10^
10C7	IgG1	+	+	–	+	+	–	2.4 × 10^−9^
11A12	IgG2a	+	+	–	+	+	–	1.4 × 10^−8^
11C5	IgG1	+	+	–	+	+	–	2.5 × 10^−10^
12H2	IgG1	+	+	–	–	–	–	ND *
13G2	IgG1	+	–	CA IV, CA IX	–	–	–	ND *
14D6	IgG1	+	+	CA II	+	+	–	2.0 × 10^−9^
15B4	IgG1	+	+	–	+	+	–	7.3 × 10^−9^
15H1	IgG1	+	+	–	+	+	–	3.3 × 10^−9^
16A8	IgG1	+	+	–	+	+	–	7.7 × 10^−10^
17E9	IgG1	+	+	–	+	+	–	1.5 × 10^−9^
17A3	IgG1	+	+	–	+	+	–	1.8 × 10^−9^
20C3	IgG1	+	+	–	+	+	–	1.6 × 10^−9^
20C4	IgG1	+	–	CA IV, CA IX	–	–	–	ND *
20G7	IgG2b	+	+	CA II, CA VI	–	–	–	ND *
22A2	IgG2b	+	+	–	+	+	–	1.2×10^−9^

* Flow cytometry non-reactive MAbs were not tested for apparent K_d_.

**Table 2:** Primers for amplification of Ig VH and VL regions. H—heavy chain, Lκ—light kappa chain.

Primer Name	Primer Sequence	Amplified Chain	Ref.
IgG1	5′–TTA ATA GAC AGA TGG GGG TGT CGT TTT GGC	H	[38]
MH1	5′–CAT ATG SAR GTN MAG CTG SAG SAG TC	H	
Kc	5′–TTA GGA TAC AGT TGG TGC AGC ATC	Lκ	
Mk	5′–CAT ATG GAY ATT GTG MTS ACM CAR WCT MCA	Lκ	
VH1FOR	5′–TGA GGA GAC GGT GAC CGT GGT CCC TTG GCC CCA G	H	[39]
VHlBACK	5′–AGG TSM ARC TGC AGS AGT CWG G	H	
VK2FOR	5′–GTT ATT TGA TCT CCA GCT TGG TCC C	Lκ	
VK1BACK	5′–GAC ATT CAG CTG ACC CAG TCT CCA	Lκ	
LB6	5′–CAT ATG ATT MAG ATR AMC CAG TC	Lκ	[40]
LB10	5′–CAT ATG ATT GWG CTS ACC CAA TC	Lκ	
LB11	5′–CAT ATG ATT STR ATG ACC CAR TC	Lκ	
LB12	5′–CAT ATG RTT KTG ATG ACC CAR AC	Lκ	
LB16	5′–CAT ATG ATT GTG ATG ACA CAA CC	Lκ	
LB17	5′–CAT ATG ATT TTG CTG ACT CAG TC	Lκ	

**Table 3:** Primers for the amplification of CA XII fragments.

Fragment Number	Amino Acid Sequence	PCR Primers	Fragments Size, kDa
#1	V27–G123	5′– ATA CATATG TTA CCCCCAGTGCAGGTG 5′– ATG CATATG GTGAACGGTTCCAAGTG	13.5
#2	Y67–F211	5′– ATT CATATG TTA GAATGCTTCCTGGCCTTTGT 5′– TTT CATATG TATGACGCCAGCCTCACG	17.7
#3	H192–S290	5′– GCC CATATG TTA GGAGAAGGAGGTGTAT 5′– GAC CATATG CATGTAAAGTACAAAGGCCA	14.3
#4	P101–S290	5′– TTT CATATG TTA GGAGAAGGAGGTGTAT 5′– ATT CATATG CCCTCGGACATGCACA	24.3
#5	F35–R220	5′– AGT CATATG TTTGGTCCTGATGGGGAGAA 5′– A GGATCC TTA GCGGTA ATATTCAGCG GTCC	23.4
#6	W43–E209	5′– GGA CATATG TGGTCCAAGAAGTACCCGTC 5′– A GGATCC TTA CT CTTCAATGTT GAATCCCG	21.2
#7	G51–N231	5′– A CATATG GGGGGCCTGC TGCAGT 5′– A GGATCC TTA GGGTTGCA AGGGGGTG	23.1
#8	D59–Y221	5′– ATT CATATG GACCTGCACAGTGACATCCT 5′– TT GGATCC TTA GTAGCGGTAATATTCAGCGG	21.1

Note: Restriction endonuclease recognition sites are underlined.

## Supplementary Materials

Supplementary Materials can be found at https://www.mdpi.com/1422-0067/21/24/9411/s1.

## Abbreviations

aa	amino acids
ADCC	Antibody-Dependent Cellular Cytotoxicity
AZM	acetazolamide
BSA	Bovine Serum Albumin
CA	carbonic anhydrase
CA IX	carbonic anhydrase IX
CA XII	carbonic anhydrase XII
CA XII^DE3^	recombinant extracellular domain of human CA XII produced in *E. coli* DE3 strain
CA XII^HEK^	recombinant extracellular domain of human CA XII expressed in human cell line HEK–293
CDR	Complementarity–Determining Regions
ELISA	Enzyme-Linked ImmunoSorbent Assay
FC	flow cytometry
FR	framework region
H	Ig heavy chain
HAMA	human anti-mouse antibody
HRP	horseradish peroxidase
Ig	immunoglobulins
IgG	immunoglobulin G
K_d_	apparent dissociation constant
L	Ig light chain
MAbs	monoclonal antibodies
OD	optical density
PBS-T	0.1% Tween-20 in PBS
PDB	Protein Data Bank
PET	positron emission tomography
PVDF	polyvinylidene difluoride
RAbs	recombinant antibodies
RIT	radioimmunotherapy
RT	room temperature
scFv	single-chain fragment variable
scFv-Fc	scFv fused to human IgG1 Fc fragment
SDS–PAG	sodium dodecyl sulfate-polyacrylamide gel
SDS–PAGE	SDS–PAG electrophoresis
TSPyV	trichodysplasia spinulosa-associated polyomavirus
V	variable region of Ig
VH	variable region of Ig heavy chain
VL	variable region of Ig light chain
VLY	vaginolysin
WB	Western blot
