# Supplementary material for: Inhibitory Monoclonal Antibodies and Their Recombinant Derivatives Targeting Surface-Exposed Carbonic Anhydrase XII on Cancer Cells
Source: Int J Mol Sci. 2020 Dec 10;21(24):9411. doi: 10.3390/ijms21249411 (PMC7763246; doi:10.3390/ijms21249411)
Supplement: Supplementary file 1 [file ijms-21-09411-s001.pdf]

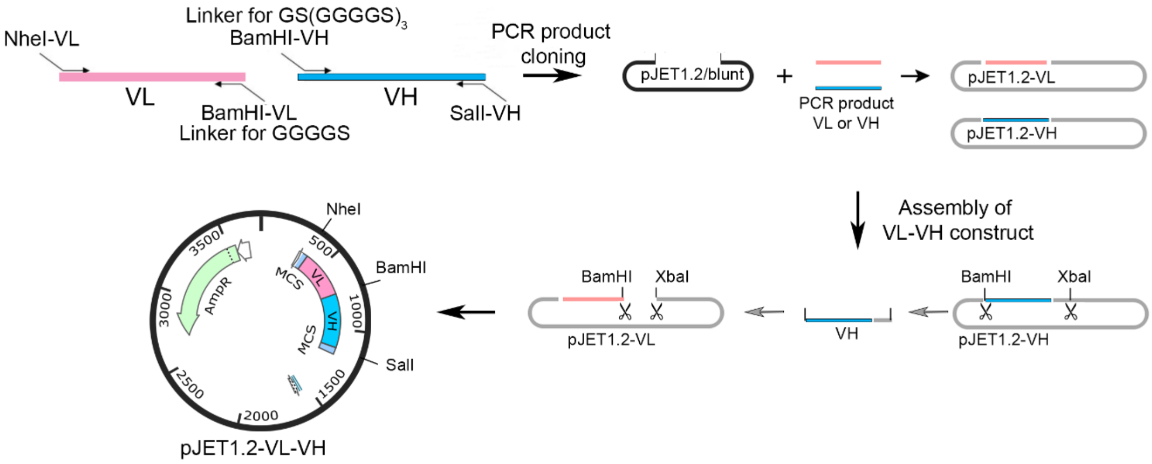

Figure S1. Schematic representation of the construction of expression vector for 14D6-derived scFv.
